# Nutrition profile and rumen fermentation of *Tithonia diversifolia* fermented with *Lactobacillus bulgaricus* at different times and doses

**DOI:** 10.5455/javar.2024.k759

**Published:** 2024-03-31

**Authors:** Roni Pazla, Novirman Jamarun, Fauzia Agustin, Arief Arief, Elihasridas Elihasridas, Ramaiyulis Ramaiyulis, Gusri Yanti, Laily Rinda Ardani, Laras Sukma Sucitra, Zaitul Ikhlas

**Affiliations:** 1 Department of Animal Nutrition and Feed Technology, Faculty of Animal Science, Andalas University, Padang, West Sumatra, Indonesia; 2 Department of Animal Production Technology, Faculty of Animal Science, Andalas University, Padang, West Sumatra, Indonesia; 3 Animal Production Technology Study Program, Agricultural Polytechnic Payakumbuh, Lima Puluh Kota, Indonesia; 4 Faculty of Social, Science and Education, Prima Nusantara Bukittinggi University, West Sumatra, Indonesia; 5 Graduate Program, Faculty of Animal Science, Andalas University, Padang, West Sumatra, Indonesia

**Keywords:** Fermentation time, inoculum dose, *Lactobacillus bulgaricus*, nutritional profile, *Tithonia diversifolia*, rumen characteristics

## Abstract

**Objective:**

This study aims to investigate the nutritional composition and rumen fermentation attributes of the tithonia plant (*Tithonia diversifolia*) treated with *Lactobacillus bulgaricus* bacteria at different fermentation durations and doses.

**Materials and Methods:**

In this research, an experimental approach employed a factorial pattern with two factors as treatments with three replications using a complete randomized design. The primary factor was the dose of *L. bulgaricus* inoculum, with concentrations at 2% and 3%. The secondary factor examined during the study revolved around the duration of fermentation, offering three time frames of 1 day, 3 days, and 5 days for analysis. The inoculum of *L. bulgaricus* contained 65 × 10^15^ CFU/ml.

**Results:**

The use of *L. bulgaricus* bacteria on tithonia plants (*T. diversifolia*) with different inoculum doses and fermentation times demonstrated a highly significant effect and significant disparities (*p* < 0.05). In phytic acid content, nutrient content (crude protein (CP), crude fiber, crude fat, and dry matter (DM)), and *in vitro* digestibility, which includes DM, organic matter (OM), CP, volatile fatty acids (VFA), NH_3_, and gas production. However, it did not show any significant interaction between pH and OM content.

**Conclusion:**

The optimal results of nutrient profiling and *in vitro* digestibility, including DM, OM, CP, rumen pH, VFA, NH^3^ (ammonia), and gas production, were observed when the tithonia plant (*T. diversifolia*) was fermented using *L. bulgaricus* with 3% inoculum doses and a fermentation time of 5 days.

## Introduction

For ruminants, forage serves as the primary source of feed. This forage feed plays a crucial role in fulfilling the fundamental life requirements of livestock, including growth, reproduction, and production [1]. When ruminants face a shortage of forage ingredients, their growth process can be hindered. Meeting the rising demand for animal protein sources and increasing livestock production will pose a significant challenge if the availability of fodder does not match the needs of the current livestock population. To overcome the scarcity of forage for livestock, one of the approaches taken involves seeking alternative feeds with high nutritional value and productivity. These alternatives should be safe for consumption by livestock, not in competition with human needs, and easily adaptable to the livestock’s diet [2].

The tithonia plant *(Tithonia diversifolia)* is one of the plants that grows wild and is commonly found at medium to high altitudes [3]. This plant shows potential as an alternative animal feed, particularly due to its high nutritional content. Several previous studies reported that the protein content of the tithonia plant was 22.98% and 18.17% crude fiber (CF) [3,4]. Tithonia plants are rich in phosphorus minerals and amino acids, which can help optimize microbial protein synthesis and microbial populations in the rumen [4]. In the previous study by Pazla et al. [4], fermenting tithonia leaves for 5 days with 3% *Lactobacillus bulgaricus* reduced the phytic acid content by 3.48 mg/100 gm with a degradation rate of 64.81% and increased digestibility up to 64%. Meanwhile, during the 7-day incubation period, using *Lactobacillus plantarum* and *Aspergillus ficuum* for tithonia fermentation was able to improve the quality of tithonia by looking at the phytase enzyme activity, feed digestibility, and rumen fermentation characteristics [4]. Meanwhile, Fasuyi et al. [5] reported that the tithonia plant *(T. diversifolia)* contains a fairly high anti-nutritional substance, phytic acid. This toxic compound can inhibit the digestive process if given to livestock. The phytic acid content of the tithonia plant is 79.2 mg/100 gm which causes a chelating taste in plants and is less palatable to livestock, so it is necessary to carry out a fermentation process to reduce the chelating taste [5]. During fermentation, the microbial dose used has a positive correlation with the increased microbial population; the dose used will affect the microbe’s ability to produce enzymes to degrade feed substrates sooner or later [5].

Fermentation is a biological process that involves microorganisms breaking down complex organic compounds into simpler compounds [6]. Fermentation using the help of microbes producing phytase enzymes is expected to reduce phytic acid levels in Tithonia plants to increase palatability [6]. In the study by Sripo et al. [7], *L. bulgaricus* is the most effective bacteria in its activity to degrade phytic acid. This microbe produces an enzyme known as phytase, specifically myo-inositol hexakisphosphate phosphohydrolase, which functions to hydrolyze phytic acid (*myoinositol hexakisphosphate*) into inorganic monophosphate. *Lactobacillus bulgaricus* was able to reduce phytic acid production in *T. diversifolia* plants by breaking phosphorus-phytate bonds [7]. The use of *L. bulgaricus* at different times and doses on tithonia (*T. diversifolia)* has not been extensively investigated. Thus, the primary aim of this investigation is to explore the nutritional profile and attributes of rumen fermentation in *T. diversifolia* when subjected to fermentation using *L. bulgaricus inoculum* at varying time intervals and doses.

## Methods and Materials

### Ethical approval

Approval from an ethical committee was unnecessary in this study since it did not involve the utilization of live animals.

### Study period and area

The experiment was conducted at the Ruminant Nutrition Laboratory, within the Faculty of Animal Science at Andalas University, located in Indonesia, during the period from June to July 2023.

### Growing inoculums of L. bulgaricus

*Lactobacillus bulgaricus* bacteria were grown on 50 ml of MRS broth media (Merck KGaA, Germany). Growth brood stock includes as much as 5 ml of *L. bulgaricus*. Incubation at 37ºC for 48 h. The inoculum of *L. bulgaricus* contained 65 × 10^15^ CFU/ml.

### Fermentation process

Tithonia leaves were dried under the sun and in the oven at 60°C for 48 h. After the tithonia plants are dry, grind them using a grinding machine, put them in a plastic storage container, and close them tightly. Tithonia plant flour was weighed at 100 gm each for 1 experimental unit, added to 160 ml of distilled water, and then placed into plastic containers for homogenization. Afterward, *T. diversifolia* was fermented using *L. bulgaricus* in 2 and 3 ml. Tightly close all samples so that no air enters. Store at room temperature for 1 day, 3 days, and 5 days according to treatment, and the samples are ready to be tested.

### Phytic acid concentration

The sample, weighing 1 gm, was mixed with 50 ml of 0.5 M HNO_3_ (Merck KGaA, Germany) and homogenized for 2 h using a shaker. The concentration of phytic acid was determined by analyzing the filter results. Filter as much as 0.5 ml mixed with 0.9 ml of 0.5N HNO_3_ and 1 ml of FeCl_3_ (containing 50 μg per ml of iron ions), covered with aluminum foil, then allow to stand for 20 min in boiling water. After cooling, 5 ml of alcohol and 1 ml of 10% ammonium thiocyanate were added to each tube, followed by centrifugation at 12.298×*g*. The measurement of absorbance at 465 nm was carried out using a UV-visible spectrophotometer (Shimadzu, Japan).

### Experimental design

An experimental approach employed a factorial pattern with two factors as treatments with three replications using a complete randomized design (CRD). The first factor was the inoculum dose of *L. bulgaricus,* which presented values of 2% and 3%. The second factor delved into fermentation time, covering periods of 1 day, 3 days, and 5 days. Tithonia’s chemical content is shown in Table 1. The treatments used were: A1B1 (2% inoculum, 1 day fermentation); A1B2 (2% inoculum, 3 days fermentation); A1B3 (2% inoculum, 5 days fermentation); A2B1 (3% inoculum, 1 day fermentation); A2B2 (3% inoculum, 3 days fermentation); and A2B3 (3% inoculum, 5 days fermentation).

### In vitro method and parameter measurements

The rumen was *in vitro* incubated using the Tilley and Terry method. A sample of 2.5 gm is put into the Erlenmeyer. To each Erlenmeyer flask, while continuously flowing with CO_2_, add 200 ml of McDougall’s buffer solution and 50 ml of rumen fluid to achieve anaerobic conditions. The tube is then closed using a rubber cover that is ventilated for gas release. After incubation, the tube was placed in a shaker water bath and incubated at 39°C for 48 h. Then, measure the rumen pH. Total gas production follows the method of Ardani et al. [8]. The supernatant results were used to analyze NH_3_ and volatile fatty acids (VFA) [9]. The residue that sticks to the filter paper is subsequently heated in the oven at 60°C for 12 h and can be used to test chemical compositions such as dry matter (DM), organic matter (OM), crude protein (CP), crude fat, and CF [9].

**Table 1.** Chemical composition of tithonia flour.

Feed ingredients	Content (%)
DM	25.57
OM	84.65
Ash	15.35
Crude fibre	22.62
Crude fat	1.92
CP	21.75
Nitrogen-free extracts	31.48
NDF	63.07
ADF	42.71
Cellulose	29.82
Hemicellulose	20.36
Lignin	7.97
Silica	4.92
Total digestible nutrient	53.52
Phytic acid	11.82 mg/100 gm

### Statistical analysis

A factorial, CRD with two factors was used in this study. Research data were run into variance analysis, and if significant differences were observed, Duncan’s test was performed for further analysis of treatment outcomes. The data analysis was done using statistical package for the social sciences (SPSS) version 26.0 (IBM Corporation, USA).

## Results and Discussion

The fermented results of tithonia plants *(T. diversifolia)* utilizing *L. bulgaricus* at varying inoculum doses and fermentation times showed the results of the nutritional profile (Table 3), *in vitro* digestibility (Table 4), and rumen fermentation characteristics (Table 5). The concentration of phytic acid is shown in Table 2.

### Phytic acid content

This study showed that the treatments had an impact (*p* < 0.05) on the content of phytic acid (Table 2). The lowest content of phytic acid was 4.30 mg/100 gm and the highest degradation was 63.62% in tithonia fermented with *L. bulgaricus* at a dose of 3% for 5 days. This can occur because *L. bulgaricus* produces phytase enzymes during the fermentation process. This is consistent with previous studies that reported that the enzyme phytase (myo-inositol-hexakisphosphate-3-phosphohydrolase) secreted by *L. bulgaricus* is an enzyme capable of catalyzing phytate *(myo-inositol hexakisphosphate)* into inorganic orthophosphate, which releases phytate-phosphorus bonds, and phosphorus can be utilized by ruminants [7,10]. This is also supported by Pazla et al. [10] *L. bulgaricus* is a lactic acid bacteria that produces phytase enzymes (*myo-inositol-hexakisphosphate).* Phosphorus is associated with the normal function of rumen microbial activity in degrading feed and is a very important factor in protein synthesis in the rumen microbial body cells [11]. Lactic acid fermentation naturally produces optimal pH conditions to be able to degrade phytate enzymes into organic phosphate and inositol forms [11]. The decrease in phytic acid content is thought to be due to the passive diffusion of phytate, which dissolves in water [12].

**Table 2.** Phytic acid concentration of *Tithonia diversifolia* fermented with *Lactobacillus bulgaricus* at different doses and fermentation times.

Nutritional profile	A factor (Dosage of *L. bulgaricus*)	B factor (fermentation time)			Average	SEM
		B1 (1 day)	B2 (3 days)	B3 (5 days)		
Phytic acid (mg/100g)	A1 (2%)	11.13^f^ ± 0.27	7.66^d^ ± 0.24	4.94^b^ ± 0.61	7.91	0.50
	A2 (3%)	10.93^e^ ± 0.13	7.11^c^ ± 0.31	4.30^a^ ± 0.81	7.45	
	Average	11.03^C^	7.39^B^	4.62^A^		

Superscript a,b,c in interaction are significantly different (*p* < 0.05). Means with different superscript in each factors are significantly different (*p* < 0.05).

**Table 3.** Nutritional profile of *Tithonia diversifolia* fermented with *L. bulgaricus* at different doses and fermentation times.

Nutritional profile	A factor (Dose of *L. bulgaricus*)	B factor (fermentation time)			Average	SEM
		B1 (1 day)	B2 (3 days)	B3 (5 days)		
DM (%)	A1 (2%)	54.78^aA^ ± 1.41	52.40^bA^ ± 1.09	50.49^cB^ ± 1.92	52.56	0.42
	A2 (3%)	54.13^aA^ ± 1.11	53.80^aA^ ± 1.59	51.75^bA^ ± 0.45	53.23	
	Average	54.46^A^	53.10^B^	51.12^C^		
OM (%DM)	A1 (2%)	86.17 ± 1.09	86.11 ± 1.31	85.42 ± 0.93	85.90	0.67
	A2 (3%)	85.87 ± 0.24	85.26 ± 1.52	84.60 ± 1.15	85.24	
	Average	86.02	85.69	85.01		
CP (%DM)	A1 (2%)	22.01^cB^ ± 0.13	24.06^bA^ ± 0.51	25.73^aA^ ± 0.46	23.93^a^	0.42
	A2 (3%)	24.99^bA^ ± 0.76	25.46^aA^ ± 1.16	25.77^aA^ ± 0.04	25.41^b^	
	Average	23.50^A^	24.76^B^	25.75^C^		
Crude fat (%DM)	A1 (2%)	1.09^aA^ ± 0.07	0.60^bA^ ± 0.03	0.51^bA^ ± 0.05	0.73	0.43
	A2 (3%)	1.07^aA^ ± 0.16	0.65^bA^ ± 0.11	0.47^bB^ ± 0.05	0.73	
	Average	1.08^A^	0.63^B^	0.49^C^		
Crude fibre (%DM)	A1 (2%)	18.82^aA^ ± 1.93	17.96^bB^ ± 0.80	17.94^bA^ ± 0.71	18.24	0.63
	A2 (3%)	19.51^aA^ ± 0.85	18.46^bA^ ± 0.50	16.91^cB^ ± 0.82	18.29	
	Average	19.17^C^	18.21^B^	17.43^A^		

Superscripts a,b,c in interaction are significantly different (*p* < 0.05). Means with different superscript in each factors are significantly different (*p* < 0.05)

**Table 4.** *In vitro* digestibility (%) of *Tithonia diversifolia* fermented with *L. bulgaricus* at different doses and fermentation times.

*In vitro* digestibility	Dose of *L. bulgaricus*	Fermentation time			SEM
		B1 (1 day)	B2 (3 days)	B3 (5 days)	
DM	A1 (2%) A2 (3%)	52.34^cB^ ± 0.02 55.10^cA^ ± 0.13	53.97^bB^ ± 0.05 58.28^bA^ ± 0.17	57.16^aB^ ± 0.08 59.12^aA^ ± 0.13	0.42
OM	A1 (2%) A2 (3%)	53.63^cB^ ± 0.28 56.38^cA^ ± 0.45	54.80^bB^ ± 0.32 59.22^bA^ ± 0.54	58.77^aB^ ± 0.26 60.13^aA^ ± 0.43	0.48
Crude fiber	A1 (2%) A2 (3%)	63.67^bB^ ± 0.24 67.00^cA^ ± 0.18	64.33^bB^ ± 0.34 70.67^bA^ ± 0.32	68.00^aB^ ± 0.38 72.00^aA^ ± 0.45	0.67

Superscripts a,b,c in different columns are significantly different (*p* < 0.05).

### Nutrition profile

The treatments on the nutritional composition of fermented tithonia impact on CP, crude fat, CF, and DM were found to be significant (*p* < 0.05), and the OM did not impact this treatment (*p* > 0.05) (Table 3). Duncan’s analysis also revealed a notable interaction between factor A and factor B. The A1B3 treatment (2% dose, 5 days of fermentation) resulted in a substantial decrease of 50.49% in the DM content. The incubation time for fermentation depends on the type of microorganism and the substrate used [12]. The occurrence of the fermentation process indicates the presence of microbial life in it. The more doses of inoculum are used, the faster the fermentation process takes place so that the amount of water released as a result of metabolism increases and the DM decreases [12]. In this study, the organic content did not exhibit any significant difference (*p* > 0.05) with respect to the variations in dose and fermentation time. The treatment has a relatively similar effect on OM content. This is assumed to be due to the phenolic compound content of Tithonia, which causes inhibition of several rumen microorganisms in breaking down feed ingredients. The rumen microbial works in the process of breaking down the substrate material to digest OM [13]. The reduction in the content of OM is due to the nutrients in the material being broken down and used by microbes. The more nutrients can be broken down due to the longer fermentation time [14].

Specifically, the A2B3 (3% dose, 5-day fermentation) demonstrated a substantial increase of 25.75%DM in CP (Table 3). This increase is related to the breakdown of inorganic nitrogen elements into cells during extended microbial growth in the fermentation process, resulting in elevated body protein content and ultimately leading to an impact on increasing CP content [14]. The high microbial population is accompanied by an increase in the content of CP, where the microbes can convert the media components into cell mass, which will form body proteins to enlarge the CP content. In the fermentation process, microbes play a crucial role by releasing enzymes, which are proteins. In addition, these microbes serve as a source of single-cell protein [15]. Increased levels of CP can also be caused by fermentation from anabolic processes that can cause microbial cell proliferation [15].

**Table 5.** Characteristics of rumen fermentation of* Tithonia diversifolia* fermented with *L. bulgaricus* at different doses and fermentation times.

Characteristics rumen fermentation	Dose of *L. bulgaricus*	Fermentation time			SEM
		B2 (3 days)	B2 (3 hari)	B3 (5 hari)	
pH	A1 (2%) A2 (3%)	6.86 ± 0.64 6.83 ± 0.56	6.87 ± 0.47 6.93 ± 0.53	6.86 ± 0.34 6.84 ± 0.51	0.04
VFA (mM)	A1 (2%) A2 (3%)	125.00^bA^ ± 0.56 126.67^cA^ ± 0.48	126.67^bB^ ± 0.39 138.33^bA^ ± 0.45	131.67^aB^ ± 0.32 145.00^aA^ ± 0.37	1.30
NH_3_ (mM)	A1 (2%) A2 (3%)	11.33^bA^ ± 0.29 12.10^cA^ ± 0.39	11.67^bB^ ± 0.45 13.90^bA^ ± 0.38	14.00^aB^ ± 0.54 14.43^aA^ ± 0.32	0.03
Total gas production (24 h)	A1 (2%) A2 (3%)	67.67^bB^ ± 2.08 82.67^cA^ ± 2.07	68.67^bB^ ± 2.02 85.67^bA^ ± 2.20	73.67^aB^ ± 2.15 87.33^aA^ ± 2.82	1.08
Total gas production (48 h)	A1 (2%) A2 (3%)	79.33^bB^ ± 3.98 112.67^ bA^ ± 3.07	91.33^aB^ ± 3.16 113.67^ bA^ ± 3.38	93.33^ aB^ ± 3.74 122.67^aA^ ± 3.20	1.5

Superscripts a,b,c in different columns are significantly different (*p* < 0.05).

Other explorations in crude fat content observed that A2B3 treatment demonstrated a decrease of 0.47%DM (*p* < 0.05) (Table 3). The results of this research are consistent with Desta et al. [15]. The impact of fermentation time on *Ensete ventricosum* contains crude fat, which is quite low at around 0.5%. Certainly, an increase in fermentation time can lead to an elevation in crude fat content, which may be attributed to the rise in short-chain fatty acids, such as hexanoic acid. The improvement in short-chain fatty acid content is due to the presence and activity of Caprociproducens species during the fermentation process [16]. This could lead to a rise in crude fat levels. The content of CF displayed significant differences (*p* < 0.05) (Table 2). A reduction in CF content was shown in the A2B3 treatment (16.91% DM) along with increasing fermentation time and fermentation dose. This experiment is in line with previous studies on decreased corn fiber in samples treated with lactic acid bacteria [16]. The content of CF in the material decreased because of the ability of microbes to carry out fiber metabolism and enzymatic damage when utilizing fiber during fermentation as a carbon source. The effectiveness of using lactic acid bacteria is indicated by a reduction in CF content accompanied by an extension in fermentation time [13,15,16].

### Digestibility of feed ingredients

The treatments had a statistically significant impact (*p* < 0.05) on the digestibility of fermented tithonia DM, OM, and CP (Table 4). Treatment A2B3 showed the highest digestibility, including DM, OM, and CP (59.12%, 60.13%, and 72.00%, respectively) compared to other treatments. Increasing the inoculum doses and extending the time of fermentation have a positive impact on improving digestibility. High doses of inoculum cause more microbial growth on the substrate and increase enzyme activity [10]. Giving feed containing high CP can increase rumen microbial activity, which will also increase the number of proteolytic bacteria and the digestibility of CP in Tithonia plants [10]. The phytase enzyme *(myo-inositol-hexakisphosphate-3-phosphohydrolase)* secreted by *L. bulgaricus* is an enzyme capable of degrading phytate *(myo-inositol hexakisphosphate)* into inorganic orthophosphate and finally releasing phytate-phosphorus bonds so that phosphorus can be utilized by ruminants [10,12]. The increasing number of microbes that grow causes more enzymes to digest feed ingredients [15]. *Lactobacillus bulgaricus* bacteria make phytase enzymes, which can reduce the levels of phytic acid present in tithonia plants. As the number of microbes increases during fermentation, there is a corresponding rise in enzyme production, which works to decrease the phytic acid content in tithonia plants [4,17].

Fermentation time is one of the determining factors; the longer the fermentation time, the more substrates will be broken down by microbial enzymes, so enzyme activity will increase [18]. The increase in DM content was due to a decrease in CF content. Increased digestibility in ruminant livestock shows that nutrients that can be broken down by microbes in the rumen are also increasing [19]. The increase in OM followed the increase in DM [19,20]. The findings of this study align with Phesatcha et al. research [21], which suggested that feed ingredients with lower fiber content are more easily digestible due to their thin cell walls, making them more susceptible to breakdown by active microorganisms in the rumen. Longer fermentation times facilitate the decomposition of CF content in the feed, resulting in an improvement in the digestibility of OM [21]. The value of CP digestibility is greatly influenced by the condition of the bacterial population in the rumen, particularly the presence of proteolytic bacteria, which are produced by extracellular protease enzymes to break down proteins and enhance the overall digestibility of CP. In line with previous research, increasing feed protein will increase NH_3_ concentrations [22].

### Characteristics rumen fermentation

The treatment applied in the study did not show a significant effect (*p* > 0.05) on the pH of the rumen (Table 5). The average value of rumen pH is in the optimal range of 6.83–6.33. These results are consistent with previous research, which also reported that the ideal pH range to facilitate digestion in the rumen was 6.8–7.0 [21]. The consistency in findings between these studies suggests that the rumen pH levels observed were conducive to effective rumen digestion. The rumen pH value of this study is suitable for the development of cellulolytic and amylolytic microbes [23]. Our exploration findings indicate that the treatment significantly influenced (*p* < 0.05) VFA production in the rumen (Table 4). Compared to the other treatments, treatment A2B3 showed the highest VFA production (145 mM). An increase in rumen fluid VFA levels indicated an improvement in OM digestibility, cellulose, and hemicellulose as a result of OM fermentation, and this is correlated with microbial rumen activity during fermentation [24]. Atasoy et al. [25] reported that the increase in the value of VFA production was due to the availability of sufficient energy and protein supplies to increase the population of rumen microbial, increasing the digestibility of the fermented material.

The study showed a significantly different effect (*p* < 0.05) on the production of NH_3_ (Table 5). Among the treatments, the A2B3 treatment displayed the highest NH_3_ production (14.43 mM) compared to the other treatments. According to Vargas et al. [23], there is a correlation between the content of protein in the ingredients and the rise in ammonia concentration in the rumen. The concentration of NH_3_ that can be used by rumen microbes depends on their growth rate and the amount of fermented protein [24]. NH3 optimum levels in the rumen ranged from 6 to 21 mM [26]. The enhancement in NH_3_ production is related to the CP content obtained in this study, thereby increasing CP digestibility and NH3 concentration due to rumen microbial degradation [27]. This study shows the impact of the treatment on total gas production (*p* < 0.05) (Table 5). Among the treatments, A2B3 exhibited the highest total gas production (122.67 ml) compared to other treatments. Gang et al. [27] found that increased DM digestibility was followed by increased gas production with the addition of lactic acid bacteria inoculum. The gas produced during rumen fermentation is considered an indicator of rumen digestibility, which is related to DM and OM digestibility [28,29]. The *in vitro* digestibility and fiber fraction content of ADF and NDF in this study, as well as total gas production, align with the reports of previous studies [29].

## Conclusion

Tithonia (*T. diversifolia*)*,* which was fermented using *L. bulgaricus* at a 3% inoculum dose and a 5-day fermentation time, gave the best results on nutrient profile, *in vitro* digestibility, including DM, OM, and CP, rumen pH, VFA, NH_3_ production, and gas production.

## List of Abbreviations

CF, crude fiber; CFU, colony-forming unit; CFU/ml, colony-forming unit per milliliter; CH_4_, methane gas; CP, crude protein; DM, dry matter; gm, grams; NH_3_, ammonia; OM, organic matter; VFA, volatile fatty acid.
